# Neutrophil Extracellular Traps as Innate Immune Reaction against the Emerging Apicomplexan Parasite *Besnoitia besnoiti*

**DOI:** 10.1371/journal.pone.0091415

**Published:** 2014-03-11

**Authors:** Tamara Muñoz Caro, Carlos Hermosilla, Liliana M. R. Silva, Helder Cortes, Anja Taubert

**Affiliations:** 1 Institute of Parasitology, Justus Liebig University Giessen, Giessen, Germany; 2 ICAAM–Instituto Ciências Agrárias e Ambientais Mediterrânicas, University of Évora, Évora, Portugal; Auburn University, United States of America

## Abstract

*Besnoitia besnoiti* infection in cattle is an important emerging protozoan disease in Europe causing economic losses and severe clinical signs, such as generalized dermatitis, orchitis, and vulvitis in affected animals. Neutrophil extracellular trap (NET) formation was recently demonstrated as an important effector mechanism of PMN acting against several invading pathogens. In the present study, interactions of bovine PMN with tachyzoites of *B. besnoiti* were investigated in this respect *in vitro*. For the demonstration and quantification of NETs, extracellular DNA was stained by Sytox Orange or Pico Green. Fluorescent illustrations as well as scanning electron microscopy analyses (SEM) showed PMN-promoted NET formation rapidly being induced upon contact with *B. besnoiti* tachyzoites. Co-localization of extracellular DNA with histones, neutrophil elastase (NE) and myeloperoxidase (MPO) in parasite entrapping structures confirmed the classical characteristics of NET. Exposure of PMN to viable, UV attenuated and dead tachyzoites showed a significant induction of NET formation, but even tachyzoite homogenates significantly promoted NETs when compared to negative controls. NETs were abolished by DNase treatment and were reduced after PMN preincubation with NADPH oxidase-, NE- and MPO-inhibitors. Tachyzoite-triggered NET formation led to parasite entrapment as quantitative assays indicated that about one third of tachyzoites were immobilized in NETs. In consequence, tachyzoites were hampered from active invasion of host cells. Thus, transfer of tachyzoites, previously being confronted with PMN, to adequate host cells resulted in significantly reduced infection rates when compared to PMN-free infection controls. To our knowledge, we here report for the first time *B. besnoiti*-induced NET formation. Our results indicate that PMN-triggered extracellular traps may represent an important effector mechanism of the host early innate immune response against *B. besnoiti* which may lead to diminishment of initial parasite infection rates during the acute infection phase.

## Introduction

Bovine besnoitiosis is an endemic disease mainly in Africa and Asia caused by the cyst-forming apicomplexan parasite *Besnoitia besnoiti*. However, upcoming with reports on *B. besnoiti* infections in Portugal in 2005 [1], there is clear evidence for a spread of this disease in Europe since outbreaks were recently also described in Spain [2], France [3], Germany [4], Italy [5], [6], [7] and Switzerland [8]. Since all these European countries had previously been free of bovine besnoitiosis the European Food Safety Authority classified this parasitosis as an emerging disease in the EU in 2010 [9].

So far, no data are available on adaptive and innate immune reactions against the apicomplexan parasite *B. besnoiti*. PMN, which are the most abundant leukocytes in the bovine blood, play a fundamental role in innate host responses since they are the earliest immune cells to arrive at the site of infection. This cell type has previously been shown to interact with both *Eimeria bovis* [10] and closely related apicomplexan parasites such as *Toxoplasma gondii* [11], [12], [13] confirming an important role of PMN in innate immune reactions against these parasites.

Besides phagocytosis and the production of antimicrobial molecules, a major effector mechanism of PMN is the formation of neutrophil extracellular traps, called NETs [14], which lead to extracellular killing of bacterial and fungal pathogens [14], [15], [16]. NET were first described by Brinkmann et al. [14] showing that PMN are capable to release granular proteins and chromatin forming thin extracellular fibers that bind Gram-positive and-negative bacteria [15], [16], [17]. The major structural component of NETs is DNA which is studded with antimicrobial proteins composed of nuclear histones, granula-derived neutrophil elastase (NE), myeloperoxidase (MPO), lactoferrin, and gelatinase [14], [18], [19]. Overall, NET formation has been described as a novel form of cell death called ETosis which is distinct from apoptosis, autophagy and necrosis and depends on the generation of reactive oxygen species (ROS) by NADPH oxidase [17], [20]. Whilst most studies have focused on bacterial and fungal pathogens, few attention has been paid on effects of NETs on apicomplexan parasites [21], [22]. Thus, NET formation has been demonstrated for *Plasmodium falciparum* [23], *E. bovis* [24] and *T. gondii* [21], [22]. In addition, NETosis was shown for different *Leishmania* species [25], [26]. The aim of this study was to describe for the first time that *B. besnoiti*-induced NET-formation which may represent an important host effector mechanism against the apicomplexan *B. besnoiti* during the acute phase of infection.

## Materials and Methods

### Host cell culture and *Besnoitia besnoiti* tachyzoite maintenance

Primary bovine umbilical vein endothelial cells (BUVEC) were isolated as previously described by Taubert et al. [27]. Briefly, umbilical cords obtained from calves born by *sectio caesarea* were kept at 4°C in 0.9% HBSS–HEPES buffer (pH 7.4; Gibco, Grand Island, NY, USA) supplemented with 1% penicillin (500 U/ml; Sigma-Aldrich, St. Louis, MO, USA) and streptomycin (500 μg/ml; Sigma). For preparation of endothelial cells, 0.025% collagenase type II (Worthington Biochemical Corporation, Lakewood, NJ, USA) was infused into the lumen of the isolated and ligated umbilical vein and incubated for 20 min at 37°C in 5% CO_2_. After gently massaging the umbilical vein, the collagenase-cell suspension was collected and supplemented with 1 ml FCS (Gibco) to inactivate the collagenase. After two washings (400×*g*, 10 min, 4°C), the cells were resuspended in ECGM (endothelial cell growth medium; PromoCell, Heidelberg, Germany), plated in 25 cm^2^ plastic culture flasks (Nunc, Roskilde, Denmark) and kept at 37°C in 5% CO_2_.

*B. besnoiti* (strain Bb1Evora04) tachyzoites were maintained by serial passages in BUVEC. Tachyzoites were collected from BUVEC supernatants, centrifuged, washed thrice with PBS, counted and suspended in RPMI 1640 medium (Gibco) until further use.

### Isolation of bovine PMN

Cattle (*n* = 3) were bled by puncture of the jugular vein. Heparinized blood was diluted in an equal amount of PBS containing 0.02% EDTA, layered on Biocoll Separating Solution (Biochrom AG) and centrifuged (800×*g*, 45 min). The pellet was suspended in 25 ml distilled water to lyse erythrocytes. Osmolarity was adjusted by adding 10× Hanks Salt Solution (HBSS, Biochrom AG). PMN were washed twice, re-suspended in RPMI medium, counted in a Neubauer haemocytometer chamber and incubated at 37°C and 5% CO_2_ for at least 30 min before use. All animal procedures were performed according to the Justus Liebig University Animal Care Committee guidelines, approved by the Ethic Commission for Experimental Animal Studies of the State of Hesse (Regierungspräsidium Giessen) and in accordance to the current German Animal Protection Laws.

### Scanning electron microscopy (SEM)

Bovine PMN were incubated with tachyzoites (ratio: 2∶1) for 10, 30, 60, and 120 min on poly-_L_-lysine (Sigma-Aldrich) pre-coated coverslips. Cells were fixed in 2.5% glutaraldehyde (Merck), post-fixed in 1% osmium tetroxide (Merck), washed in distilled water, dehydrated, critical point dried by CO_2_-treatment and sputtered with gold. Specimens were examined using a Philips XL30 scanning electron microscope at the Institute of Anatomy and Cell Biology, Justus Liebig University Giessen, Germany.

### Quantification of NETs

NET formation was quantified using PicoGreen (Invitrogen). Therefore bovine PMN (*n* = 3) were incubated with tachyzoites (2∶1 ratio) for different time spans (30–300 min, 37°C). In order to estimate the effects of the parasite viability or integrity on NET formation, tachyzoites were either attenuated by UV-light according to Zhao Y et al., 2013 [28] 60 min, 230 V/50–60 Hz), heat-inactivated (60°C, 30 min) or homogenized [three freeze and thaw cycles plus sonication (15 s, 50 kHz, ice bath)] (1∶1 ratio; 5×10^5^, 90 min, *n* = 3). To evaluate dose-dependent effects different PMN:tachyzoites ratios were used (1∶1, 1∶2, 1∶3). For positive controls, zymosan (Invitrogen) was used (1 mg/ml). To estimate maximum values of extracellular DNA, PMN were lysed by Triton-X 100 treatment (0.1%; Sigma-Aldrich). To block NET formation, 90 U of DNase I (Roche Diagnostics) were used. NET inhibition assays were performed using diphenylene iodonium (10 μM, Sigma-Aldrich). After PMN/parasite co-cultures, micrococcal nuclease was added (5 U/well, New England Biolabs) (15 min, 37°C). Afterwards samples were centrifuged (300×*g*, 5 min). The supernatants were transferred (100 μl per 96-well) and PicoGreen (50 μl/well, diluted in 10 mM Tris/1 mM EDTA) was added. NET-formation was determined by spectrofluorometric analysis (484 nm excitation/520 nm emission) using an automated reader (Varioskan Flash; Thermo Scientific).

### Visualization of NETs and detection of histones (H3), neutrophil elastase (NE) and myeloperoxidase (MPO) in *Besnoitia besnoiti* tachyzoites-induced NET structures

After incubation of bovine PMN with tachyzoites (ratio 1∶1, 60 min) on poly-_L_-lysine-treated coverslips and fixation of the samples [4% paraformaldehyde, Merck], NET structures were visualized by staining extracellular DNA with Sytox Orange (Invitrogen) according to Martinelli et al. [29] and Lippolis et al. [30]. For the visualization of tachyzoites within NET structures, tachyzoites were stained with CFSE (7.5 μM, 37°C, 30 min; Invitrogen) according to Hermosilla et al. [31] prior to PMN confrontation.

After fixation and three washings in PBS, samples were mounted in anti-fading buffer (Mowiol, Sigma-Aldrich). For the detection of histones, MPO and NE within NET structures the following antibodies were used: anti-histone (H3) monoclonal (DyLight, ab139848, Abcam], anti-MPO (Alexa Fluor 488, ABIN906866, Antibodies-online.com) and anti-NE (AB68672, Abcam) antibodies. Samples were washed thrice, blocked with BSA (1%, Sigma-Aldrich) and incubated in antibody solutions [1 h, room temperature (RT), for anti-histone; 24 h, RT, for anti-MPO and anti-NE antibodies]. The samples were washed in PBS and mounted in anti-fading buffer). Visualization was achieved using an invert Olympus IX81 fluorescence microscope.

### Estimation of ROS, MPO and NE activities

ROS production was measured by oxidation of DCFH-DA (Sigma-Aldrich) to fluorescent DCF according to Conejeros et al. [32], [33]. PMN (*n* = 3) were re-suspended in HBSS containing Ca^2+^ and incubated with *B. besnoiti* tachyzoites at 37°C in a 1∶1 ratio (2.5×10^5^ cells/well) in duplicates for 30 min of exposure. Thereafter, DCFH-DA (10 μg/ml) was added to each duplicate. For positive controls zymosan was used (1 mg/ml). The relative fluorescence units (RFU) were recorded at 15 min intervals for a period of 120 min applying 485 nm excitation and 530 nm emission wavelengths.

For the measurement of MPO activity, Amplex red reagent (Invitrogen) was used for peroxidase activity assessment. PMN and tachyzoites (1∶1 ratio, *n* = 3) were incubated (30 min, 37°C) in HBSS-buffer without phenol red (Gibco). For positive controls zymosan was used (0.5 mg/ml). After incubation, 50 μM Amplex red was added to each well and peroxidase activity was measured every 10 min for 1 h in 571–585 nm fluorescence ranges.

NE activity was evaluated using the chromogenic substrate MeoSuc-Ala-Ala-Pro-Val-chloromethyl-ketone (Sigma-Aldrich). PMN (*n* = 3) were exposed to tachyzoites (1∶1 ratio, 30 min, 37°C). For positive controls zymosan was used (1 mg/ml). After incubation, chromogenic substrate (3 mg/ml) was added to each sample just prior to measurement. NE activity was measured at 410 nm wavelength using an automated reader (Varioskan Flash; Thermo Scientific. NE- and MPO-inhibition assays were performed using the NE inhibitor chloromethyl ketone (CMK, 1 mM Sigma-Aldrich) and the MPO inhibitor ABAH (100 μM, Calbiochem) according to Parker et al. [34]. PMN were pre-incubated with the corresponding inhibitor (30 min, RT) prior to exposure to viable tachyzoites (1∶1 ratio, 30 min, RT). Thereafter, NET formation was analyzed as described above.

### Tachyzoite entrapment assay

Tachyzoite entrapment was quantified according to Nizet et al [35]. PMN were pre-activated by zymosan treatment (1 mg/ml, 30 min, 37°C). Meanwhile, tachyzoites were stained with CFSE (7.5 μM, 37°C, 30 min; Invitrogen) and washed twice in PBS. Thereafter, zymosan-stimulated PMN were exposed to CFSE-labeled tachyzoites (30 min, 37°C) in ascendant ratios (1∶2; 1∶3). Non-pre-exposed tachyzoites were used for controls. For inhibition, diphenylene iodonium (DPI; 10 μM) was added to PMN 30 min prior to exposure to CFSE-labeled tachyzoites as control of NET inhibition. The samples were washed twice in RPMI and measured for fluorescence intensities at 485/538 nm wavelengths. The percentage of entrapment was calculated as follows: [(A485/538 nm tachyzoites exposed to PMN)/(A485/538 nm non-exposed tachyzoites)] ×100%.

### Host cell invasion assay

To test for the effect of parasite-triggered NETs on tachyzoite infectivity, three different experimental setups were chosen: 1) tachyzoites were incubated with PMN (1∶2 ratio, 3 h, 37°C) allowing for effective NET formation. 2) For comparative reasons, an equal number of tachyzoites used in the setup 1 that were not pre-exposed to PMN were incubated in plain medium. 3) Similar to setup 1, equal numbers of tachyzoites were incubated with PMN (1∶2 ratio, 3 h, 37°C) allowing for effective NET formation. Additionally, to resolve potential NET structures, DNase (90 U/well) was added 15 minutes before the end of the incubation period. In the next step, tachyzoites of setups 1–3 were transferred to confluent BUVEC (one 25 cm^2^ flask for each setup) as host cells and incubated (1 h, 37°C, 5% CO_2_). Overall three different BUVEC isolates were used in this experiment. After incubation, BUVEC layers were washed to remove PMN and dead/excrescent tachyzoites. Infection rates were estimated microscopically 24 h p. i. in ten randomly selected vision power fields (400× magnification).

### Statistical analysis

By using normal distribution of data, co-culture/stimulation conditions were compared by one- or two-factorial analyses of variance (ANOVA) with repeated measures. Differences were regarded as significant at a level of *p*≤0.05.

## Results

### Tachyzoites of *Besnoitia besnoiti* exposed to bovine PMN trigger NET formation

**Figure 1:**
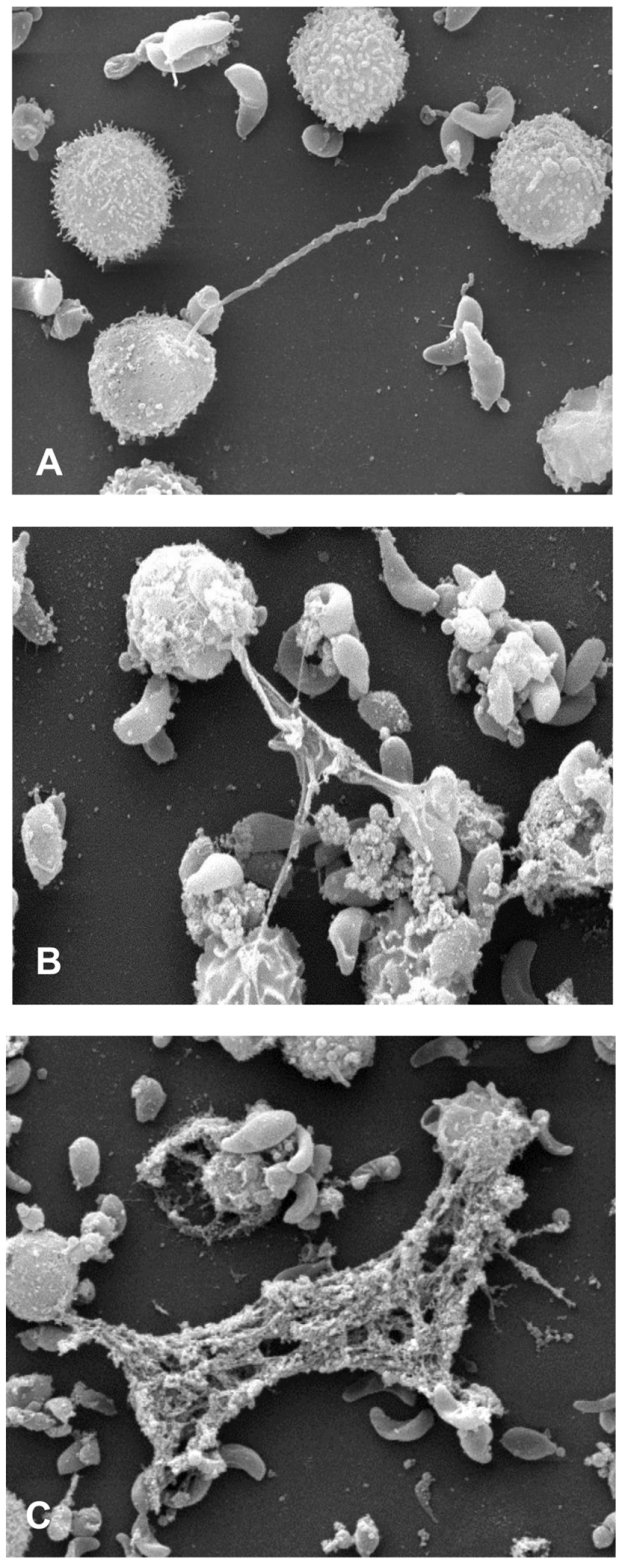
NETs formed by bovine PMN after confrontation with *B. besnoiti* tachyzoites. Scanning electron microscopy analysis revealed NETs being formed by bovine PMN co-cultured with *B. besnoiti* tachyzoites for different time periods [(A) 10 min (B) 30 min, (C) 60 min] in the absence of serum. (A) Delicate PMN-derived filaroid structure being attached to a tachyzoite, (B) Several tachyzoites being trapped in a network of long drawn-out fibres originating from dead and disrupted PMN. (C) Conglomerate of tachyzoites and a rather chunky meshwork of PMN-derived filaments.

SEM analyses revealed that exposure of live *B. besnoiti* tachyzoites to bovine PMN resulted in the formation of a delicate network of thicker and thinner strands of fibres originating from PMN and being firmly attached to the parasites, seemingly trapping them (Fig. 1). Kinetic analyses revealed different degrees of NETosis: after 10 min of exposure delicate PMN-derived filaroid structures being attached to tachyzoites were detected (Fig. 1A). Here, PMN still exhibited the morphology of intact cells. Later on, tachyzoites being trapped in a network of long drawn-out fibres originating from disrupted PMN (Fig. 1B, 30 min) and conglomerates of tachyzoites and rather chunky meshworks of PMN-derived filaments (Fig. 1C, 60 min) were observed.

**Figure 2:**
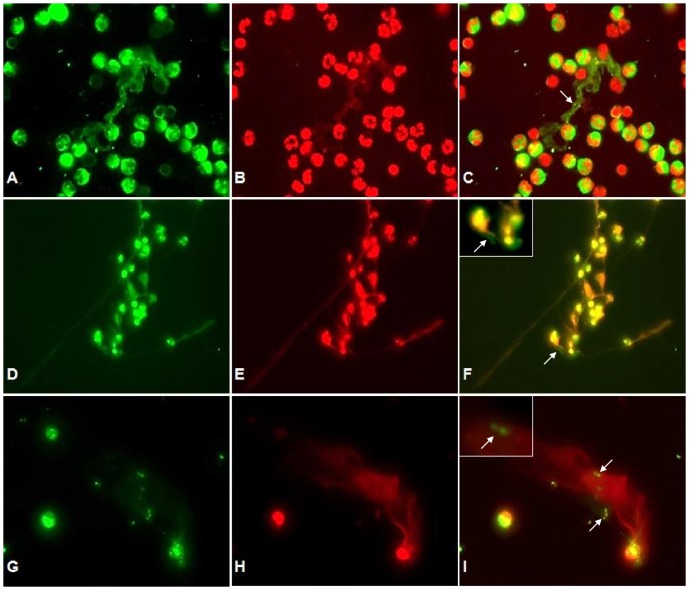
Co-localization of DNA with histones (H3), NE and MPO in tachyzoite-induced NET structures. Co-cultures of bovine PMN and *B. besnoiti* tachyzoites were fixed, permeabilized, stained for DNA using Sytox Orange (red: B, E, H) and probed for MPO (green: A), histones (green: D) and NE (green: G) using anti-MPO, anti-histone (H3) and anti-NE antibodies and adequate conjugate systems. Areas of respective co-localization (merges) are illustrated in C, F, I. The arrow in (C) indicates delicate globular structures within NETs. Arrows in (F) and (I) indicate tachyzoites being trapped in NET structures. Photomicrographs are of representative cells from 3 independent experiments. The time culture in this experiment was 60 min.

These parasite-induced NET-like structures were proven to contain DNA by Sytox Orange staining (Fig 2B, E, H). Tachyzoites were found in close proximity to NETs and presumably were trapped in these structures (Fig. 2F, I). Furthermore, co-localization of extracellular DNA with H3 (histone H3), NE and MPO in parasite entrapping structures confirmed the classical characteristics of NETs (Fig. 2). Furthermore, we observed the moment of NET extrusion of a NE-granule positive PMN capturing tachyzoites in NETs being decorated with NE-positive material (Fig. 2G–I).

**Figure 3:**
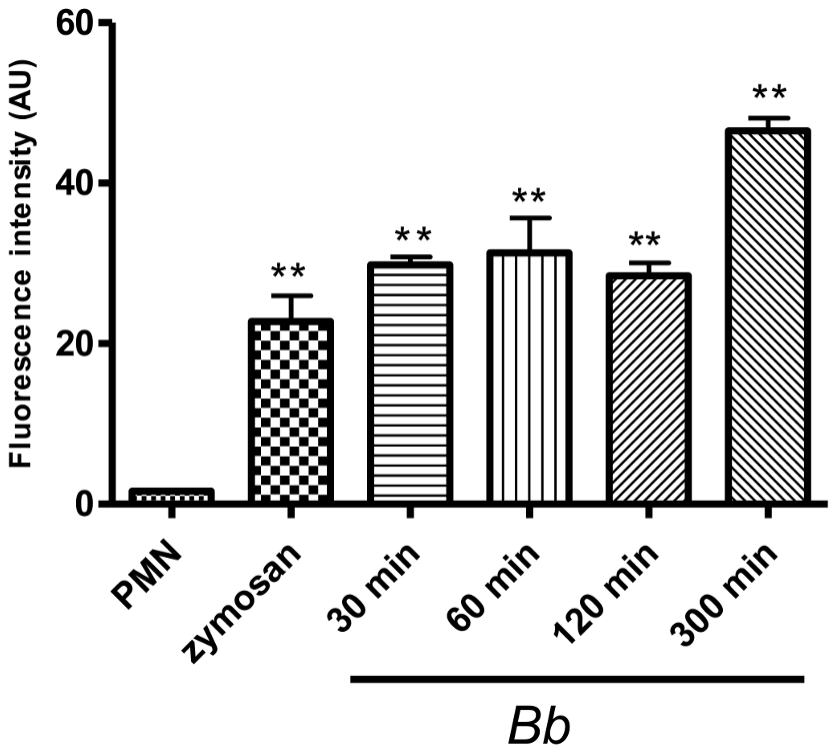
Kinetics of tachyzoite-triggered NET formation. PMN were incubated with *B. besnoiti* tachyzoites (ratio 2∶1; 4×10^5^ PMN: 2×10^5^ tachyzoites), zymosan (1 mg/ml, positive control) or plain medium (negative control) for different time periods. After incubation, samples were analysed for extracellular DNA by quantifying PicoGreen-derived fluorescence intensities. Each condition was performed in triplicates. Arithmetic means of three PMN donors, minimum and maximum. Differences were regarded as significant at a level of *p*≤0.05.

**Figure 4:**
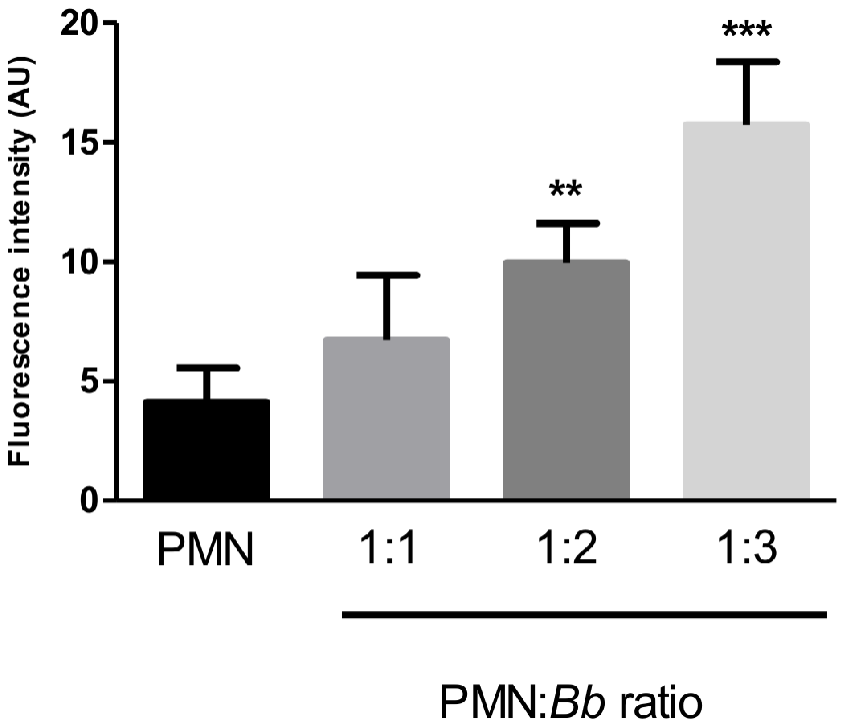
Dose-dependency of tachyzoite-triggered NET formation. PMN and *B. besnoiti* tachyzoites were incubated at different ratios (PMN:tachyzoites = 1∶1, 1∶2, 1∶3). After incubation, samples were analysed for extracellular DNA by quantifying PicoGreen-derived fluorescence intensities. Each condition was performed in duplicates. Arithmetic means of three PMN donors, minimum and maximum. Differences were regarded as significant at a level of *p*≤0.05.

Quantification of fluorescence intensities mirroring NET formation revealed that exposure of PMN to *B. besnoiti* tachyzoites significantly increased the amount of extracellular DNA when compared to parasite-free controls (*p*<0.01; Fig. 3). Furthermore, parasite-induced NET formation was dose-dependent, as increasing the amount of tachyzoites led to enhanced fluorescence intensities (Fig. 4).

Kinetic studies quantifying NET formation revealed fast and strong NET induction. Thus, strong reactions were observed already after 30 min of exposure, i. e. at the earliest time point measured in this assay. Notably, the values for NET formation were higher than those of the positive control after all time points tested indicating the strong capability of *B. besnoiti* tachyzoites to trigger NETosis. Given that Triton X100-treatment reflected lysis of all PMNs ( = 100%), co-cultures of PMN and tachyzoites at a ratio of 1∶2 led to 76.4±2.03% DNA release of the PMN, respectively. In contrast, in parasite-free negative controls 4.03±0.33% of the PMN contributed to extracellular DNA content of the samples (data not shown).

**Figure 5:**
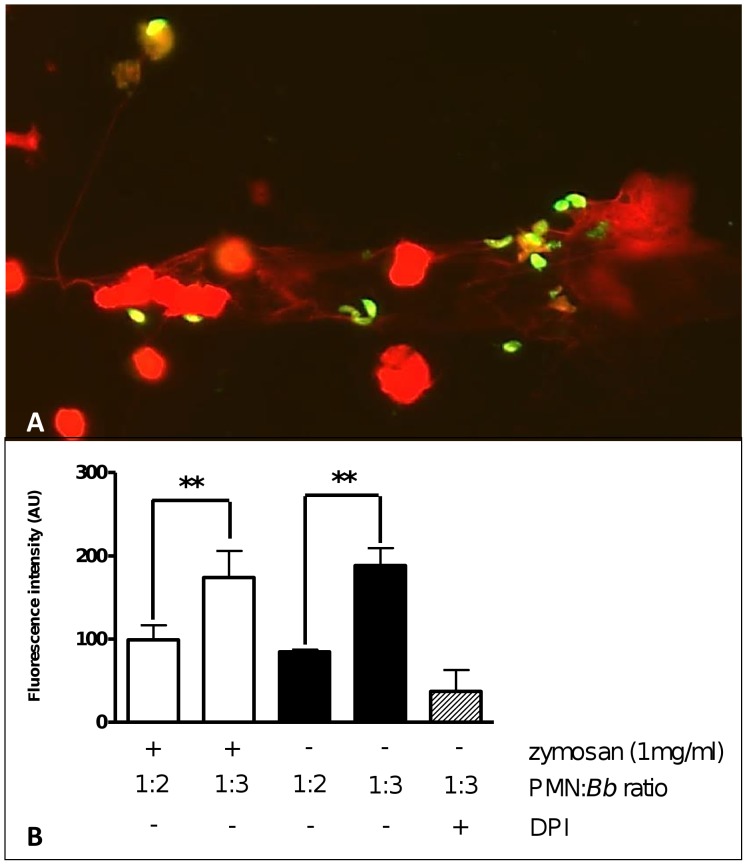
Quantification of *B. besnoiti* tachyzoite entrapment in NET structures. Entrapment of *B. besnoiti* tachyzoites within NET structures was illustrated after the exposure of CFSE- (7.5 μM, 37°C, 30 min) stained tachyzoites to bovine PMN and a subsequent DNA-staining by Sytox Orange (A). Quantification of tachyzoite entrapment in NETs (B) was performed after incubation of non-stimulated and zymosam-pre-activated PMN (*n* = 3; 2×10^5^/96-well) with CFSE-stained tachyzoites (7.5 μM, 37°C, 30 min) for 30 min at ratios of 1∶2 and 1∶3. Thereafter, non-trapped tachyzoites were washed off and the resulting fluorescence intensities were calculated in relation to non-exposed CFSE stained tachyzoites. As an inhibition control DPI (10 μM) treatment was used. All experiments were performed as duplicates. Differences were regarded as significant at a level of *p*≤0.05.

Since parasite entrapment was observed in SEM analyses, we established quantitative parasite-entrapment-assays using CFSE-stained parasites. Thus we could illustrate tachyzoite entrapment within NET structures (Fig. 5A). Furthermore, NET-formation led to a dose-dependent parasite capture revealing up to 34% of tachyzoites being immobilized in NET structures. (Fig. 5), when a ratio of 3∶1 (tachyzoites:PMN) was applied. However, pre-activation of PMN did not significantly alter NET formation after tachyzoite exposure (Fig. 5). In order to validate that parasite entrapment was caused by NET formation, PMN were preincubated with DPI (10 μM). As expected, a diminishment of fluorescence intensity derived from CFSE-stained parasites was observed.

### Tachyzoite-triggered NETosis is accompanied by up-regulation of ROS, NE and MPO activities in PMN

**Figure 6:**
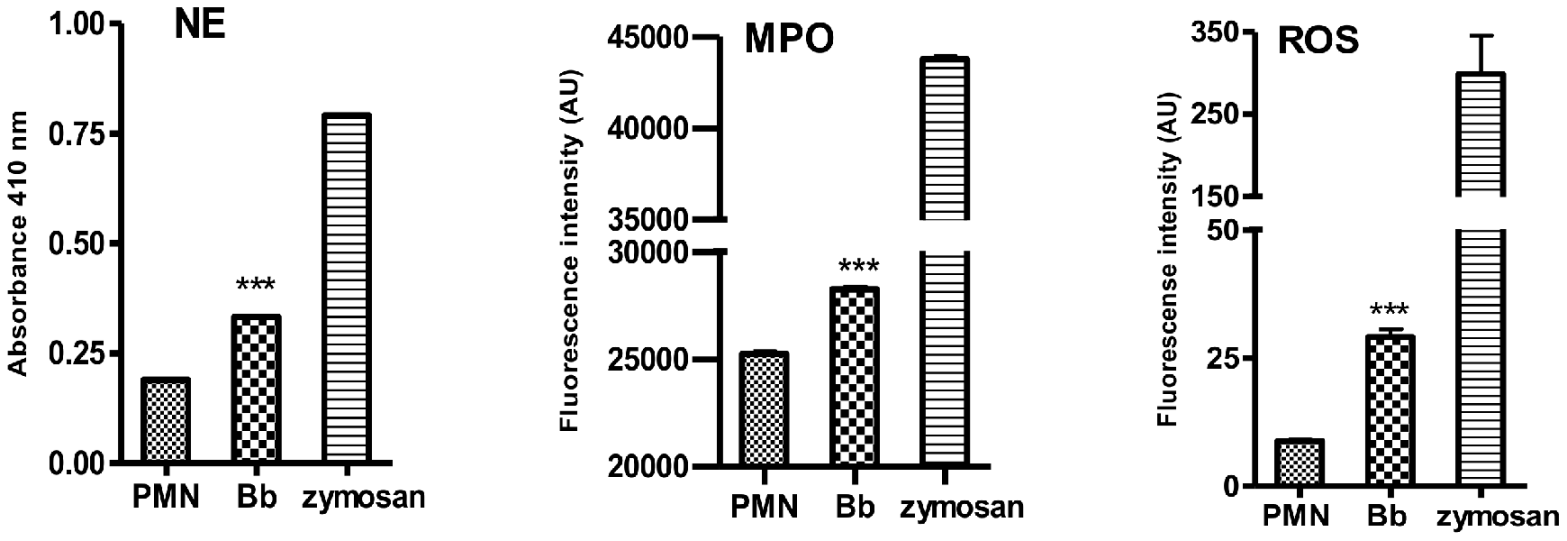
ROS production and enzymatic activities of NE and MPO in tachyzoite-exposed bovine PMN. Bovine PMN were exposed to *B. besnoiti* tachyzoites, zymosan (positive control) or plain medium (negative control) for 30 min. Thereafter, enzymatic activities of NE and MPO as well as ROS production were measured in the supernatants via the NE-chromogenic substrate MeoSuc-Ala-Ala-Pro-Val-chloromethyl ketone, Amplex red and the oxidation of DCFH-DA to fluorescent DCF, respectively. All experiments were performed in triplicates. Arithmetic means of three PMN donors, minimum and maximum. Differences were regarded as significant at a level of *p*≤0.05.

Stimulation with zymosan serving as positive control significantly enhanced the enzymatic activities of NE as well as MPO, and of the ROS production in PMN (Fig. 6). Furthermore, exposure of bovine PMN to tachyzoites significantly induced NE and MPO enzymatic activities and ROS production (*p*<0.001), indicating these molecules as key factors in tachyzoite-induced NET formation.

### Tachyzoite-triggered NET formation is diminished by treatments with DNase and inhibitors of NADPH oxidase, NE and MPO

**Figure 7:**
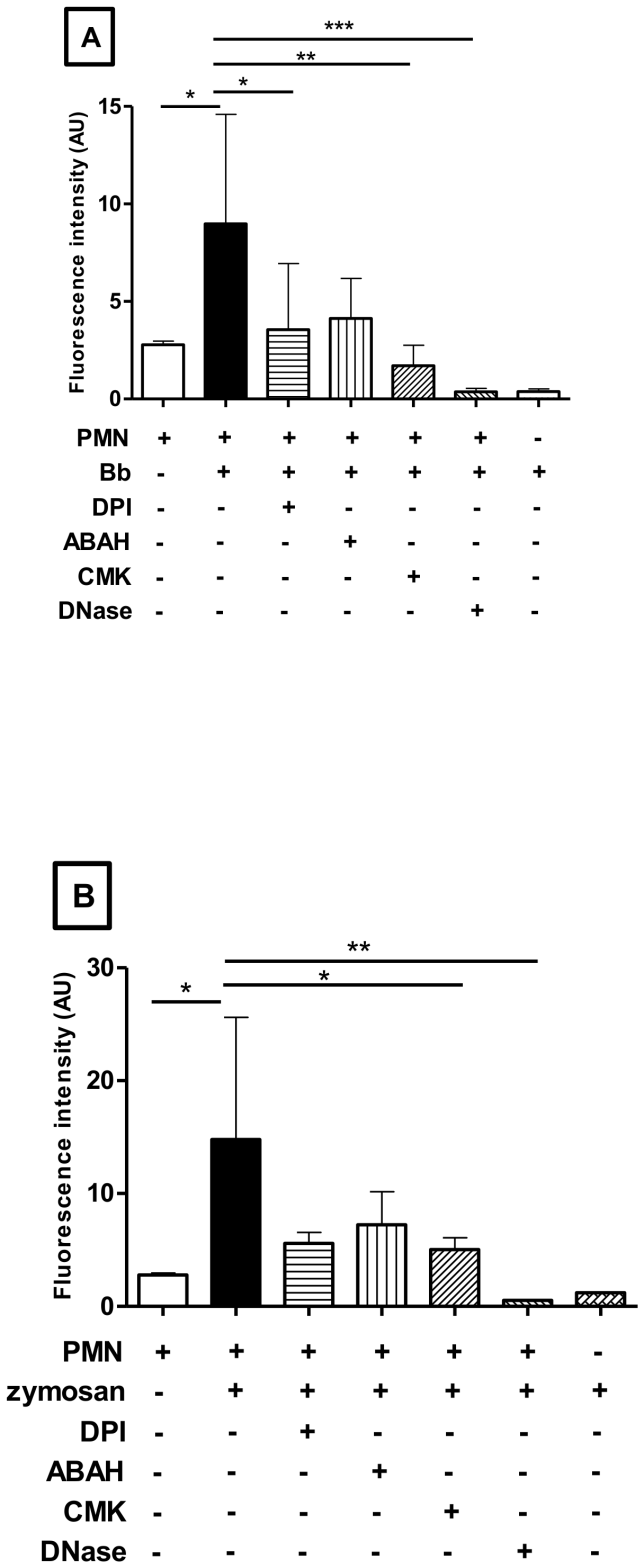
Inhibition of *B. besnoiti* tachyzoite-triggered NET formation. Fig. 7A: PMN were exposed to *B. besnoiti* tachyzoites in the presence or absence of inhibitors and DNase I (90 U). Cells were pre-incubated with DPI (10 μM), NE inhibitor (CMK, 1 mM) and the MPO inhibitor ABAH (100 μM) prior to exposure to tachyzoites (1∶1 ratio, 30 min, RT). After an incubation period of 30 min with *B. besnoiti* tachyzoites NET-formation was determined by quantifying PicoGreen-derived fluorescence intensities (484 nm excitation/520 nm emission). The same experiment was performed with zymosan as positive control (1 mg/ml; Fig. 7B). Plain medium was used as negative control. Each condition was performed in triplicates for each PMN donor (*n* = 3). Differences were regarded as significant at a level of *p*≤0.05.

The DNA-nature of *B. besnoiti*-induced NET-like structures was additionally confirmed by DNase treatment (Fig. 7A). A significant reduction of PicoGreen-derived fluorescence intensities after co-culture with tachyzoites was measured in DNase-treated samples (*p*<0.001). To further confirm the characteristics of NETs we performed inhibition assays with DPI, an inhibitor of the NADPH oxidase. Supplementation of DPI throughout the incubation period resulted in a significant reduction of parasite-induced NET formation (*p*<0.05; Fig. 7A). In addition, pre-incubation of PMN with NE and MPO inhibitors (CMK and ABAH respectively) resulted in a significant decrease of tachyzoite-triggered NET formation (p<0.01; Fig. 7A). In order to confirm NET characteristics after zymosan treatment, the experiment was performed with the positive control (zymosan, 1 mg/ml; Fig. 7B) and as expected we observed significant diminishment of NET formation after treatment with all inhibitors mentioned.

### Tachyzoite-induced NET induction only marginally depends on the parasite's integrity or viability

**Figure 8:**
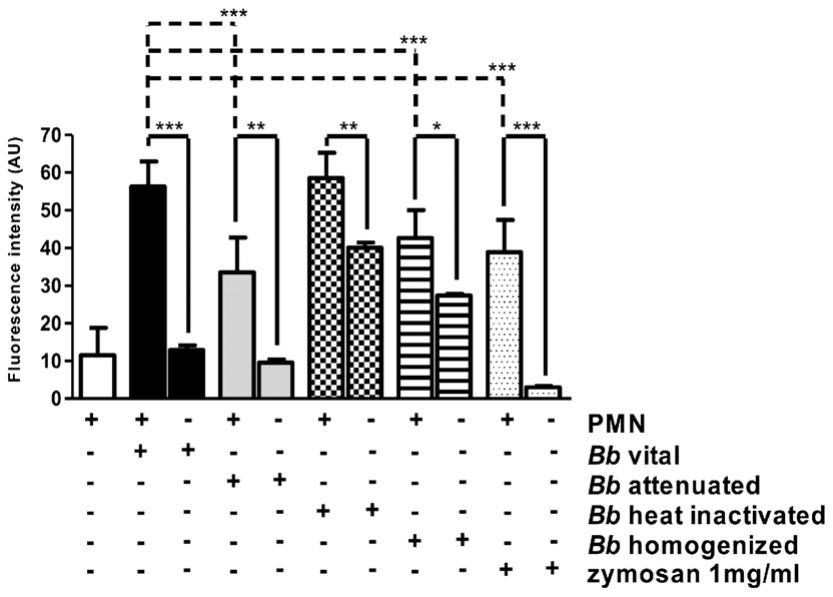
NET formation induced by differentially treated tachyzoites. Bovine PMN were exposed to vital, UV- attenuated, homogenized, and heat-inactivated *B. besnoiti* tachyzoites for 90 min. Stimulation with zymosan (1 mg/ml) served as positive control, plain medium was used as negative control. Samples were analysed for extracellular DNA by quantifying Pico Green-derived fluorescence intensities. Each condition was performed in triplicates. Arithmetic means of three PMN donors, minimum and maximum. Differences were regarded as significant at a level of *p*≤0.05.

To analyze the role of tachyzoite viability and integrity in parasite-induced NETosis experiments were performed using either viable, attenuated (via UV-irradiation), dead (via heat-inactivation) or crushed (via homogenization) tachyzoites. Overall, all different tachyzoite formulations significantly induced NET formation when compared to non-exposed PMN (*p*<0.001; Fig. 8). Moreover, significant differences concerning NET formation were observed between all differentially treated tachyzoites exposed to PMN and the respective controls (vital *p*<0.001; attenuated *p*<0.01; heat inactivated *p*<0.01; homogenized *p*<0.05). However, diminished reactions were driven by homogenized parasites exposed to PMN. Consequently, these reactions resulted in significant differences compared to NET formation induced by vital tachyzoites (*p*<0.001; Fig. 8). It is noteworthy, that in case of heat-inactivated and homogenized parasites the DNA background in the controls was rather high and most probably resulted from free tachyzoite DNA. These overall results indicate that *B. besnoiti*-induced NET formation is at least partially dependent on the tachyzoite integrity.

### Parasite-induced NET formation prevents tachyzoites from invading host cells

**Figure 9:**
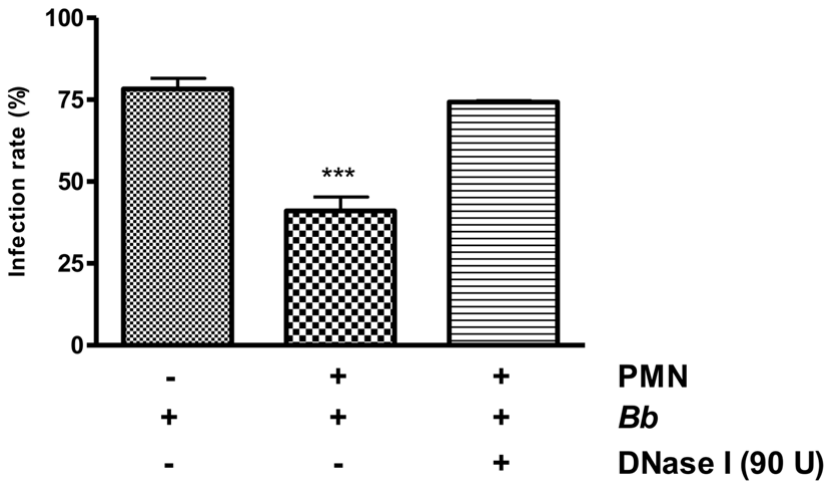
Infectivity of *B. besnoiti* tachyzoites after exposure to bovine PMN. Vital *B. besnoiti* tachyzoites were co-cultured for 3 h with bovine PMN ( =  PMN + *B.b*.) allowing for effective NET formation. To dissolve potential NET structures, DNase was supplemented 15 min before the end of the incubation period ( =  *B.b.* + PMN + DNase). Incubation of tachyzoites in plain medium served as PMN-free, infection control ( =  *B.b.* only). After incubation, samples were transferred to confluent BUVEC monolayers for 1 h. Thereafter, the cell layers were thoroughly washed and infection rates were estimated. Arithmetic means and standard deviations of three PMN donors, minimum and maximum. Differences were regarded as significant at a level of *p*≤0.05.

Host cell invasion is an indispensable requirement for successful survival and replication of the obligate intracellular parasite *B. besnoiti*. To determine the NET-triggered parasite-entrapment on subsequent tachyzoite infectivity, PMN-exposed tachyzoites were transferred to BUVEC as host cells and infections rates estimated thereafter. In parallel equal numbers of tachyzoites that had not been exposed to PMN before were used for BUVEC infection. As shown in Fig. 9, previous encounter of tachyzoites with PMN and, most probably, subsequent NET formation, significantly prevented the parasites from active invading host cells afterwards. Thus, infection rates decreased from 78.3±3.24%, resulting from non-exposed tachyzoites, to 41.06±4.26% achieved by PMN-pre-exposed tachyzoites, i. e. NET formation hampered tachyzoites from host cell invasion and led to a 40% reduction of the infection rate which may have a tremendous impact on subsequent parasite proliferation. To prove that this impairment was owed to NET formation, potential NETs were dissolved via DNase treatment being performed after 165 min of PMN-tachyzoite-exposure (i. e. after a time period that allowed for efficient NET formation) and such treated tachyzoites (used in equal numbers to the other setups) were used for BUVEC infection. As depicted in Figure 9, the infectivity of tachyzoites was completely restored by this treatment (74.27±0.25% infection rate) proving that *i*) the ensnarement of tachyzoites in NET structures hampered a large proportion of tachyzoites from host cell invasion, and *ii*) that NETs were not able to exhibit killing activities on tachyzoites within a time period of 3 hours.

## Discussion

The results of this study show that bovine PMN strongly release NETs in response to the tachyzoite stage of *B. besnoiti*. The data emphasize the relevance of this effector mechanism in the defense of *B. besnoiti* as parasite-triggered NET formation actively interferes with host cell invasion of tachyzoites thereby abrogating their further development which is ultimately linked to an intracellular lifestyle.

Since the first description of NETs as innate effector mechanism in 2004 [14], most studies focused on the effect of NETs on bacterial and fungal pathogens. However, there is increasing evidence on the relevance of NETosis as defense mechanism against protozoan infections. To date, NET-related data are only available on some apicomplexan species (*T. gondii*, *Plasmodium falciparum*, *E. bovis*) and *Leishmania* spp. [22], [23], [24], [25], [26]. With *B. besnoiti* we add a new apicomplexan parasite which is of high importance for cattle industry as envisioned by the declaration as emerging disease in the EU through the European Food Safety Authority in 2010 [9]. PMN-derived NET structures being firmly attached to *B. besnoiti* tachyzoites and subsequently leading to parasite entrapment were visualized by SEM as well as fluorescence imaging analyses. As equally described for *E. bovis* sporozoites [24] and *T. gondii* tachyzoites [22], quantitative assays revealed fast and strong induction of NETs by *B. besnoiti* tachyzoites. Overall, up to 76% of PMN were found to be involved in NET formation when compared to Triton-X lysis of PMN. In contrast to *E. bovis* sporozoites [24], tachyzoite-triggered NETs did not exhibit a clear time-dependency and induced reactions of almost equal strength irrespective of the time of incubation. Since other works reported on remarkable quantitative differences in extent and time course of pathogen- and phorbol 12-myristate 13-acetate (PMA) induced NET formation [17], [24] and since PMA does not exhibit proper activation capacity on bovine PMN in contrast to human cells [36], we used zymosan for the stimulation of NETosis by bovine PMN in positive controls. In contrast to PMA, this molecule was demonstrated as potent activator of PMN in the bovine system [36]. Overall, stimulation of bovine PMN with zymosan turned out as reliable positive control inducing ROS-, NE- and MPO-activities as well as NET release.

NETs mainly consist of chromatin [14]. Thus, we confirmed the DNA-nature of tachyzoite-triggered NET by staining with Sytox Orange/PicoGreen. In addition, the resolution of parasite-induced NETosis by DNase treatments proved this typical characteristic of NETs. Besides chromatin/DNA, the major components of NETs are nuclear histones and granular components such as NE, MPO, lactoferrin, and gelatinase [14], [18], [19]. These molecules are of high relevance concerning the microbiocidal mechanism of NETs [14], [37], [38], [39]. Applying co-localization analyses concerning extracellular DNA and histones (H3), NE or MPO in tachyzoite-entrapping structures we confirmed these classical characteristics of NETs. Furthermore, NE and MPO inhibitors treatments significantly reduced NETs formation in tachyzoite-exposed PMN revealing the essential role of these enzymes in *B. besnoiti*-induced NETosis.

The process of NET formation depends on the assembly/activation of the NADPH oxidase complex resulting in ROS production [40], [17]. As reported for several other pathogens [14], [17], [22], [24], *B. besnoiti*-triggered NET production also proved to be NADPH oxidase-dependent since it was significantly diminished by DPI treatment. Furthermore, the relevance of this enzyme complex in NET formation was confirmed by enhanced ROS production of tachyzoite-exposed PMN.

Parasite entrapment in NETs proved to be dose dependent. Overall, a third of tachyzoites were ensnared by NET structures when applying a ratio of 3∶1 (tachyzoites: PMN). As previously reported for *E. bovis* sporozoites [24] NET-mediated parasite entrapment had an enormous implication on the infectivity of tachyzoites as shown in functional infection experiments. Thus, infection rates dropped dramatically when tachyzoites were pre-exposed to bovine PMN prior to endothelial host cell encounter, indicating that NETs were hampering the parasites from active host cell invasion. As expected, DNase treatment completely resolved this effect. This result furthermore suggested that NETs do not exhibit detrimental/lethal effects on tachyzoites as has been proposed for some bacterial pathogens. Since *B. besnoiti* is an obligate apicomplexan intracellular protozoan and since pathogenicity of the parasite is ultimately linked to continuous infection and proliferation cycles in endothelial cells *in vivo* [9], [41] NET-mediated parasite entrapment and inhibition of host cell invasion will surely have an impact on the outcome of the disease.

So far, NET-triggering molecules originating from apicomplexan parasites are not known. To evaluate the influence of the parasites viability and/or integrity we used different formulations of tachyzoites. Overall, morphologically intact parasites (vital and UV-irradiated) all significantly triggered NET release in bovine PMN with the latter treatment leading to significantly reduced reactions. In contrast to *E. bovis* sporozoites [24], tachyzoites of *B. besnoiti* induced NETs irrespective of their viability. However, in accordance to *E. bovis* sporozoites [24] and *T. gondii* tachyzoites [22], soluble parasite lysates also induced significant NET release in PMN. In case of *B. besnoiti*, these reactions were significantly weaker than those induced by vital tachyzoites but still significant when compared to the negative control.

The results of this study demonstrated for the first time *B. besnoiti* tachyzoites as a strong inducer of NET formation. Considering the life cycle of *B. besnoiti* which includes active proliferation in endothelial cells during the acute phase of the disease, parasite entrapment via NET formation may be of particular importance *in vivo* since lysis of infected endothelial cells will lead to direct exposure of tachyzoites to blood components, such as circulating PMN and other leukocytes. It is worth noting that *B. besnoiti*-infected bovine endothelial cells display increased adhesion molecules gene transcription and enhanced PMN adhesion (Taubert, personal observation) allowing for close proximity of effector cells and parasites. However, so far the role of NET formation in *B. besnoiti*-infected cattle *in vivo* is not clear and will be difficult to determine. Nevertheless out data suggest parasite-induced NET formation as an effective and important effector mechanism in host innate immune reactions directed against *B. besnoiti*.
